# Expression of epithelial-Mesenchymal transition related markers in Plasmacytoid Urothelial carcinoma of the urinary bladder

**DOI:** 10.1186/s12894-020-00641-2

**Published:** 2020-06-22

**Authors:** Shunichiro Nomura, Yasutomo Suzuki, Jun Akatsuka, Yuki Endo, Akira Shimizu, Tsutomu Hamasaki, Go Kimura, Yukihiro Kondo

**Affiliations:** 1 Department of Urology, Nippon Medical School, 1-1-5 Sendagi, Bunkyo-ku, Tokyo, 113-8603 Japan; 2 Analytic Human Pathology, Nippon Medical School, 1-1-5 Sendagi, Bunkyo-ku, Tokyo, 113-8603 Japan

**Keywords:** Bladder, Urinary bladder, Plasmacytoid, Urothelial carcinoma, E-cadherin, Snail, Epithelial-mesenchymal transition

## Abstract

**Background:**

Plasmacytoid urothelial carcinoma (PUC) of the urinary bladder is a variant of urothelial carcinoma that carries a poor prognosis. The epithelial-mesenchymal transition (EMT) has been demonstrated to contribute to tumor progression. As the cause of the increased aggressiveness of PUC is unknown, we investigated PUC and EMT-related marker expression.

**Methods:**

A total of 633 bladder carcinoma cases diagnosed from 2006 to 2015 at the Nippon Medical School Hospital were analyzed. Twelve patients were found to have plasmacytoid histology and diagnosed with PUC. Slides were evaluated for percentage of plasmacytoid variant, and stained for E-cadherin, N-cadherin, Vimentin, Fibronectin and Snail expression.

**Results:**

The incidence of PUC was 1.9% (12/633). The median patient age at diagnosis was 71 years (range, 60–80 years) and the male-female ratio was 11:1. All but three patients had stage T2b or higher. The median overall survival was 10 months. In 10/12 cases, Snail and N-cadherin were positive. Vimentin was positive in 9/12 cases. Fibronectin was positive in 8/12 cases. While E-cadherin was negative in 10/12 cases. Nine cases showed > 10% plasmacytoid component. Eight of the nine patients (88.9%) with > 10% plasmacytoid component died.

**Conclusions:**

The results indicate that PUC may induce EMT and may be associated with high invasion.

## Background

Plasmacytoid urothelial carcinoma (PUC) of the urinary bladder was first reported in 1991 as a similar histological type to plasma cell [1]. The World Health Organization (WHO) working group classified PUC as a variant of urothelial carcinoma (UC). These tumor cells have eosinophilic cytoplasm and eccentrically placed, enlarged hyperchromatic nuclei with small nucleoli [2]. The prognosis of PUC of the bladder is worse than that of conventional UC [3]. However, why PUC is more aggressive is unknown.

Later inquire about proposes that the epithelial-mesenchymal transition (EMT) is a vital figure related to tumor progression and metastasis [4, 5]. EMT could be a handle at first watched in embryonic improvement in which cells lose epithelial characteristics and pick up mesenchymal properties to extend motility and invasion [4]. Besides, a later ponder detailed that Snail could be a key controller of EMT [6]. Snail is a superfamily of zinc-finger transcription factors that was, to begin with distinguished in *Drosophila melanogaster* [7]. Snail actuates EMT, in portion, by straighforwardly repressing epithelial markers such as E-cadherin and by upregulating mesenchymal markers such as N-cadherin, Vimentin, and Fibronectin. Immunohistochemistry has appeared downregulated or negative E-cadherin expression in the majority of PUC [3, 8]. Hence, PUC may actuate EMT. Therefore, EMT may be associated with PUC progression. The present study examined the expression status of EMT-related markers (E-cadherin, N-cadherin, Vimentin, Fibronectin and Snail) in PUC.

Whether survival is related to the proportion of plasmacytoid variant histology is unknown. Thus, we assessed the association between the proportion of plasmacytoid variant histology and survival in PUC patients. Furthermore, we also report clinical outcome information.

## Methods

### Patients and samples

The cohort under investigation comprised 12 patients who had bladder carcinoma with plasmacytoid histology at our institution between March 2006 and August 2015. All hematoxylin and eosin stained glass slides were retrieved and reviewed to confirm the diagnosis using the WHO definition of “plasmacytoid variants” [2]. Having been compiled for research purposes, this group represents patients for whom pretreatment archival paraffin-embedded tissue blocks and data from complete clinical follow-up were available. Tumors were graded histologically in accordance with WHO classifications and were staged as per the TNM staging system of the Union for International Cancer Control (2009). The amount of PUC as a percentage was evaluated in the transurethral resection of bladder tumor (TURBT) and compared with that in the cystectomy when available.

### Immunohistochemistry

Immunostaining was performed on at least one representative paraffin section using routine laboratory standard protocols. The antibodies used on paraffin-embedded tissues included EMA (Dako, Glostrup, Denmark), CK7 (Dako), CK20 (Dako), E-cadherin (Nichirei, Tokyo, Japan), N-cadherin (TaKaRa, Otsu, Japan), Vimentin (Dako), Fibronectin (Abcam, Cambridge, UK), Snail (Abcam) and CD138 (Dako). The stained tumor tissues were evaluated blindly with respect to clinical patient data. Staining was assessed using a semiquantitative scoring system (0, 1+, 2+, and 3+). Immunohistochemical staining was evaluated as follows: 0, no staining of tumor cells; 1+, faint staining in less than 10% of tumor cells; 2+, weak or moderate staining in more than 10% of tumor cells; and 3+, strong staining in more than 10% of tumor cells. Staining intensity of 0 or 1+ was considered negative, while 2+ or 3+ staining was considered positive. Negative controls were incubated without the primary antibody.

### Statistical analysis

Associations between E-cadherin and IHC characteristics of PUC were analyzed using the Fisher’s exact test. *P*-values < 0.05 were considered significant. All statistical analyses were performed with SPSS Version 21.0 statistical software package (IBM Corp, Armonk, NY, USA).

## Results

### Clinical data

Among the 633 patients with bladder carcinoma that were initially identified, 12 were classified as PUC. Tumors diagnosed as PUC comprised 1.9% (12/633) of all bladder carcinomas. The clinicopathologic features of the 12 patients are shown in Table 1. The median patient age at diagnosis was 71 years (range, 60–80 years) and there was only one female patient.

**Table 1:** Clinical and pathologic features of 12 cases of plasmacytoid urothelial carcinoma

Case	Presenting	TNM	Pathologic	Plasmacytoid
No.	Symptoms	Clinical Stage	T stage	Component (%)
1	Hematuria	T3aN0M0	2b	> 10
2	Hematuria	T1N0M0	1	> 10
3	Hematuria	T2bN0M0	2b	> 10
4	Hematuria	T3bN0M0	3b	> 10
5	Frequent urination	T4aN0M0	4a	> 10
6	incidental	T1N0M0	1	> 10
7	Residual urine	T3bN0M0	2b	< 10
8	Hematuria	T3bN2M0	2b	> 10
9	Hematuria	T2bN1M0	2b	> 10
10	Hematuria	T1N0M0	1	< 10
11	Hematuria	T2bN0M0	2b	> 10
12	Hematuria	T2bN0M0	2b	< 10

TNM stage was cT1N0 in three patients, cT2bN0 in three, cT3b-4aN0 in four, cT2bN1 in one, and cT3bN2 in one. The initial diagnosis of plasmacytoid carcinoma of the bladder was made on TURBT in 11 cases and cystoprostatectomy in one case. The  pathologic T stage was pT1 in three patients, pT2b in seven, pT3b in one, and pT4a in one. None of the 12 cases had a prior history of bladder cancer.

The treatment outcomes of the 12 patients are shown in Table 2. One patient underwent radical cystectomy preceded by neoadjuvant chemotherapy. However, this surgical specimen was not pathologically free of cancer (pT2<). One patient underwent radical cystectomy alone, and two patients underwent palliative cystectomy. In all available specimens from patients who underwent surgery, the negative margin and negative lymph node status rate at surgery were 75% (3 of 4) and 0% (0 of 2). Three patients received chemotherapy alone, and one of the three patients remained alive with disease and the other patient died of disease. All chemotherapy regimen was GC (gemcitabine and cisplatin). One patient received chemoradiotherapy alone and one patient received intravesical bacillus Calmette-Guerin immunotherapy alone. Two patients rejected any further treatment after TURBT. One patient did not receive any further treatment after TURBT due to a rapid worsening cancer.

**Table 2:** Treatment outcomes in 12 patients with plasmacytoid histology

Case		Post-treatment	Follow-Up	
No.	Treatment	Progression site	Months	Status
1	Radical cystectomy	Pelvic	7	Died of disease
2	Chemotherapy	Retroperitoneal lymph nodes	10	Died of disease
3	TURBT	Bladder	9	Died of disease
4	Radical cystectomy	Lung	5	Died of disease
5	Chemoradiation	Retroperitoneal lymph nodes	9	Died of disease
6	Chemotherapy alone	No progression	35	Alive with disease
7	TURBT	No progression	36	Alive with disease
8	Radical cystectomy and Chemotherapy	No progression	11	Died of chemotherapy
9	Chemotherapy	Retroperitoneal lymph nodes	3	Died of disease
10	BCG	No progression	30	Alive with disease
11	TURBT	Retroperitoneal lymph nodes	3	Died of disease
12	Radical cystectomy	Retroperitoneal lymph nodes	15	Died of disease

At the time of analysis in August 2015, disease progression was objectively documented in 8 of the 12 patients. Disease recurrence was observed in the retroperitoneal lymph nodes in 5 of these 12 patients (42%), while 1 (8%) had recurrence in lung, and 1 patient each showed recurrence in bladder or pelvis.

Eleven patients had follow-up information available, while one was lost to follow-up. Eight of the patients died of their disease from 3 to 15 months (median 9 months), while three patients were alive from 29 to 36 months (median 36 months). One patient death was attributed to chemotherapy. With a median follow-up of 9.5 months, the overall median survival was 10 months. The 1-year survival rate was 33.3% for all patients.

Cases were stratified based on the percentage of plasmacytoid variant histology. Nine cases (75%) showed > 10% plasmacytoid component, while three cases (25%) showed < 10% plasmacytoid component. Eight of the nine (88.9%) patients with > 10% plasmacytoid component died. The 1-year survival of patients was 11.1% (8/9 cases) for > 10% plasmacytoid patients. Conversely, the 1-year survival of patients was 100% (3/3 cases) for < 10% plasmacytoid patients.

### Immunohistochemistry

**Fig. 1:**
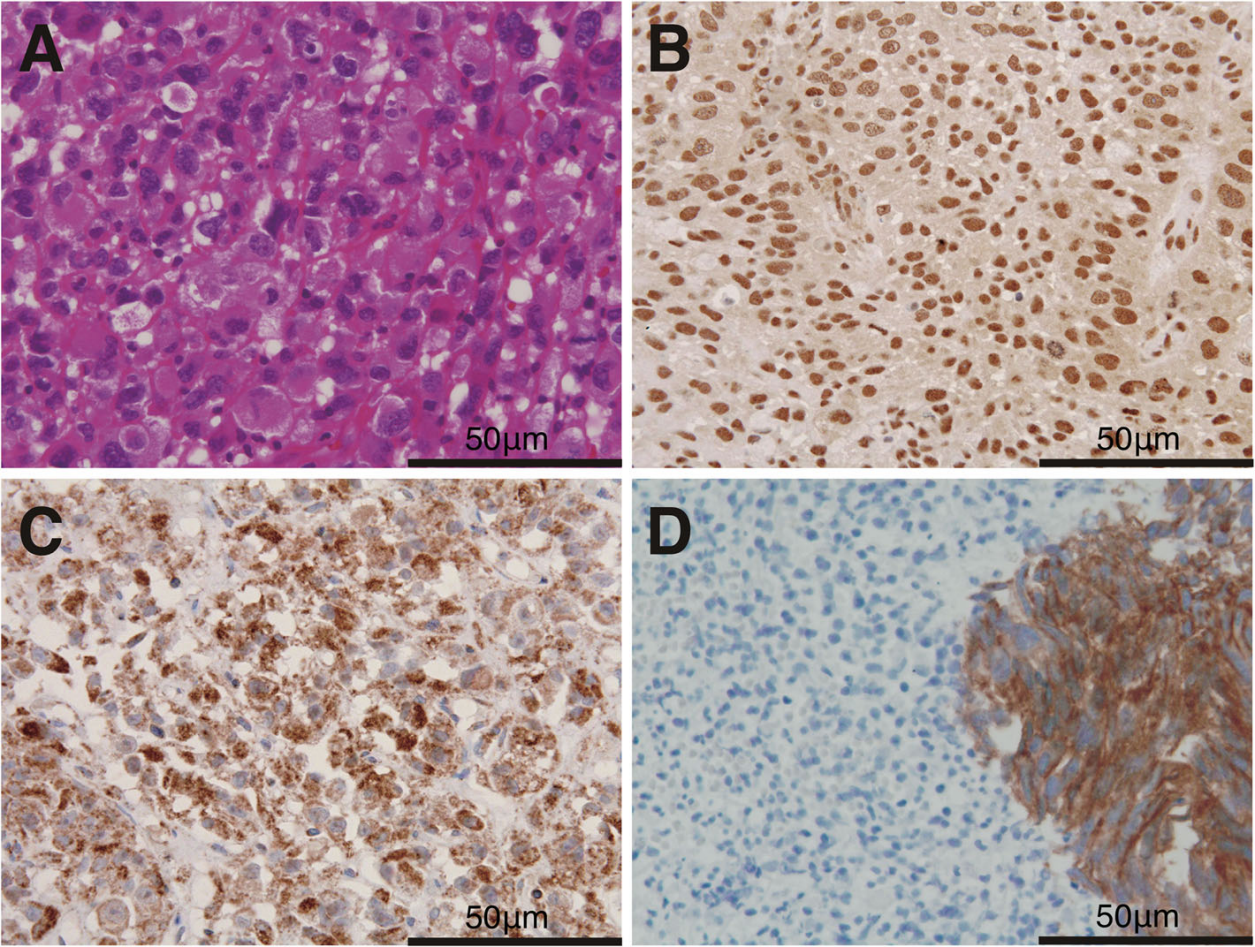
**a.** Hematoxylin and eosin staining: the tumor cells have eosinophilic cytoplasm and eccentrically placed, enlarged hyperchromatic nuclei with small nucleoli. **b.** Snail-positive tumor cells. **c.** N-cadherin-positive tumor cells. **d.** E-cadherin-negative tumor cells of PUC with an E-cadherin-positive typical urothelial carcinoma

The immunohistochemical findings in the 12 cases are summarized in Table 3. EMA was positive in 5/12 cases (41.7%), CK7 was positive in 7/12 cases (58.3%), CK20 was positive in only 2/12 cases (16.7%). E-cadherin was negative in 10/12 cases (83.3%). N-cadherin and Snail were positive in 10/12 cases (83.3%). Vimentin was positive in 9/12 cases (75%). Fibronectin was positive in 8/12 cases (66.7%). There was no difference in these EMT-related markers expression between E-cadherin positive and negative PUC cells (Table 4). CD138 was negative in all cases. Snail was localized in the nucleus of PUC cells. E-cadherin and N-cadherin were localized in the cytoplasm of PUC cells. Typical UC was positive for E-cadherin. Representative cases of immunohistochemical staining are presented in Fig. 1.

**Table 3:** Summary of the immunostaining results

Case No.	EMA	CK7	CK20	E-cadherin	N-cadherin	Vimentin	Fibronectin	Snail	CD138
1	–	–	–	–	+	+	+	+	–
2	+	–	–	–	+	+	+	+	–
3	–	–	–	–	+	+	+	+	–
4	–	+	–	–	+	+	+	+	–
5	+	+	–	+	+	+	+	+	–
6	–	+	+	+	+	–	–	+	–
7	+	+	–	–	+	+	–	+	–
8	–	–	–	–	–	+	–	+	–
9	+	+	–	–	+	+	+	+	–
10	–	–	–	–	–	–	–	–	–
11	–	+	–	–	+	+	+	+	–
12	+	+	+	–	+	–	+	–	–

**Table 4:** IHC characteristics of PUC stratified according to the presence of E-cadherin

	EMA neg.	CK7 neg.	CK20 neg.	N-cadherin pos.	Vimentin pos.	Fibronectin pos.	Snail pos.
E-cad. Neg.	6/10	5/10	9/10	8/10	8/10	7/10	8/10
	60%	50%	90%	80%	80%	70%	80%
E-cad. Pos.	1/2	0/2	1/2	2/2	1/2	1/2	2/2
	50%	0%	50%	100%	50%	50%	100%
*P* Value	1	0.47	0.318	1	0.455	1	1

## Discussion

PUC is recognized as a rare and aggressive variant of UC, which often presents at a high stage and carries a poor prognosis [3]. Sahin et al. first described this tumor in 1991 [1]. Mai et al. reported an incidence of 2.7% of PUC in a series of muscle invasive UC [9]. In this study, 9 of 12 tumors were invasive. 9 of 12 PUC patients died during follow-up. The median age at diagnosis was 71 years, a finding similar to other recent reports. We recognized that the poor prognoses of these cases are due to the high invasion and the high clinical stage at presentation [3]. However, it is unclear how often PUC shows high invasion.

EMT is an essential step amid epithelial tumor metastasis [10]. EMT causes changes in cell-cell and cell-extracellular matrix interactions coming about in transmigration of cancer cells, in this way driving to metastasis [11, 12]. E-cadherin may be a cell-cell intersection protein that is regularly downregulated or misplaced amid EMT, while expression of N-cadherin, Vimentin, Fibronectin, and Snail are obtained amid this process. In this study, E-cadherin expression was largely negative, while N-cadherin, Vimentin, Fibronectin and Snail were mostly positive. The present study reported a possibility of EMT in PUC.

E-cadherin is a key structure protein in maintaining both the stability of adhesion between epithelial cells and the stability of tissues [13]. Recent studies have demonstrated that loss of E-cadherin expression may correlate with high grade and advanced stage of UC [14, 15]. Other studies reported that the majority of PUC cases show low E-cadherin expression [3, 8]. Keck et al. reported that most PUC with loss of membranous E-cadherin show a nuclear accumulation of E-cadherin. E-cadherin also serves as an independent prognostic factor for reduced overall survival of patients with muscle-invasive bladder cancer who were treated with radical cystectomy and adjuvant chemotherapy [16]. In this study, 10 of 12 cases (83.3%) showed largely negative membranous E-cadherin.

Snail is considered a fundamental controller of EMT and, thus, of tumor movement. Bruyere et al. detailed that Snail expression predicts tumor recurrence in superficial bladder cancer [17]. Kosaka et al. reported that Snail expression might be a prognostic indicator of disease-free survival and disease-specific survival in upper urinary tract UC [18]. We moreover already detailed that Snail expression may anticipate poor results in bladder cancer patients treated with neoadjuvant chemotherapy [19]. In this study, Snail was mostly positive in 83.3%. PUC also may predict the poor outcome by Snail. Further studies with a large cohort of PUC patients are needed to confirm this result.

Fibronectin is an essential component of extracellular matrix. It has been found highly expressed in a few kinds of cancer, indicating a potential role in progression [20, 21]. Malmstrom et al. reported the level of urine fibronectin in bladder cancer patients were significantly higher than that in patients with benign urothelial diseases and the health groups [22]. Furthermore, muscle invasive bladder cancer patients have a significantly higher level of urine fibronectin [23]. In this study, fibronectin was all negative in the patient had no progression. Although our study is the small number of cases, fibronectin may predict the recurrence of PUC patients.

CD138 expression has been reported as a specific marker for PUC. However, Goto et al. reported the frequency of CD138 positivity in PUC was relatively low, compared with that observed in the conventional types and other variants [24]. In this study, CD138 was negative in all cases. Therefore, PUC should be correctly diagnosed using their characteristic cytomorphology.

Whether survival is related to the proportion of plasmacytoid variant histology has been unknown. Here we assessed the association between the proportion of plasmacytoid variant histology and survival in PUC patients. Analysis of the correlation between the amount of PUC and outcome revealed that the three patients with < 10% plasmacytoid component did not die from cancer, while eight of the nine patients (88.9%) with > 10% plasmacytoid component died from disease. This result may demonstrate the importance in identifying the amount of PUC.

It remains unknown how PUC patients should be treated. Qiang et al. reported PUC was not associated with worse overall mortality compared with pure UC on multivariable analysis in a large cohort of patients treated with radical cystectomy [25]. Radical cystectomy may improve survival for patients with PUC. In this study, only four PUC patients underwent radical cystectomy and three patients underwent only TURBT. Radical cystectomy should be considered as the first line choice for PUC.

## Conclusions

Our study is the first to elucidate immunohistochemical evidence of EMT in PUC. PUC may induce EMT and may be associated with high invasion. More detailed studies are needed to address this question.

## Data Availability

The datasets during and/or analyzed during the current study available from the corresponding author on reasonable request.

## Abbreviations

PUC: Plasmacytoid urothelial carcinoma
WHO: World Health Organization
UC: Urothelial carcinoma
EMT: Epithelial-mesenchymal transition
TURBT: Transurethral resection of bladder tumor
